# Conscientious objection to referrals for abortion: pragmatic solution or threat to women’s rights?

**DOI:** 10.1186/1472-6939-15-15

**Published:** 2014-02-26

**Authors:** Eva M Kibsgaard Nordberg, Helge Skirbekk, Morten Magelssen

**Affiliations:** 1 Centre for Medical Ethics, Institute of Health and Society, University of Oslo, Pb. 1130 Blindern, N-0318 Oslo, Norway; 2 Norwegian Advisory Unit for Learning and Mastery in Health, Oslo University Hospital, Oslo, Norway; 3 Department of Behavioural Sciences in Medicine, Institute of Basic Medical Sciences, Faculty of Medicine, University of Oslo, Oslo, Norway

**Keywords:** Abortion, Conscientious objection, General practitioner, Patient rights

## Abstract

**Background:**

Conscientious objection has spurred impassioned debate in many Western countries. Some Norwegian general practitioners (GPs) refuse to refer for abortion. Little is know about how the GPs carry out their refusals in practice, how they perceive their refusal to fit with their role as professionals, and how refusals impact patients. Empirical data can inform subsequent normative analysis.

**Methods:**

Qualitative research interviews were conducted with seven GPs, all Christians. Transcripts were analysed using systematic text condensation.

**Results:**

Informants displayed a marked ambivalence towards their own refusal practices. Five main topics emerged in the interviews: 1) carrying out conscientious objection in practice, 2) justification for conscientious objection, 3) challenges when relating to colleagues, 4) ambivalence and consistency, 5) effects on the doctor-patient relationship.

**Conclusions:**

Norwegian GP conscientious objectors were given to consider both pros and cons when evaluating their refusal practices. They had settled on a practical compromise, the precise form of which would vary, and which was deemed an acceptable middle way between competing interests.

## Background

Some health professionals object to induced abortion on moral or religious grounds. Conscientious objection involves a potential conflict between the interests of health professionals on the one hand, and the interests of patients and society on the other. Objections to referrals for abortion pit the physician’s need not to be involved in the process leading up to abortion against the duty to ensure continuity of care.

Norwegian health professionals (physicians, nurses and midwives) have a right to refrain from performing and assisting in induced abortion (Abortion act § 14). Only one other conscience right is legally recognized, to refrain from participating in assisted reproduction. The right to conscientious objection does not cover referrals for abortion by general practitioners (GPs). However, ever since the Abortion act was passed in 1978 some GPs have silently refused to refer for abortions. It is not known how many of Norway’s 4300 GPs object to referrals for abortion. An official nationwide survey found that 16 GPs objected. However, this survey had a low response rate [1].

In Norway abortions are performed in public hospitals and paid for in full by the state. Current Norwegian legislation and public health policy provides for abortion on demand in the first 12 weeks of gestation. Between the 13^th^ and 18^th^ week, abortion is allowed by committee approval on medical, eugenic, criminal, humanitarian, or social grounds. After the 18^th^ week, abortion is only allowed in special circumstances. The current law was passed with a one-vote majority in 1978, after heated debates. In 1974, both "The Women’s Campaign for Abortion on Demand" and the "Popular Movement against Abortion on Demand" were formed. Feminists and Christians, respectively, formed the bases for the campaigns. Attitudes in the Norwegian general population changed from 47% supporting abortion on demand in 1974, to 58% in 1981 [2] and 76% in 2010 [3]. The medical community was split in two. Today there is little debate about the current law.

In the Norwegian ‘regular GP scheme’ each citizen is assigned a particular GP by the municipality. Patients can choose to switch GPs twice a year. However, many Norwegian municipalities are sparsely populated, some encompassing less than 1000 inhabitants with only one or two GPs. Fears have therefore been raised that refusals to refer may impede availability of abortion.

Yearly, about 15000 abortions are carried out. In Norway it is typically GPs, not gynecologists, who refer for abortions. There are about 4300 Norwegian GPs, implying that each GP on average sees 3–4 patients seeking abortion yearly.

The practice of refusing to refer for abortion has gained the attention of the regulatory bodies on a few occasions. These have stated that the practice may be acceptable, depending on the municipality’s ability to organize services so that the patient’s right to health care is met. However, this changed when an October 2011 regulation explicitly outlawed conscientious objection by GPs in any context. In January 2014, the new coalition government (Conservative Party/Progress Party) announced new legal regulations which would allow conscientious objection by GPs in the specific context of referrals for abortion only [4]. Parliamentary debate is expected in the fall of 2014. The development of the regulation of conscientious objection to referrals for abortion, then, has gone through three stages: first toleration in a legal vaccum; then outlawed by 2011 regulations which are still valid; finally, the most likely outcome is a renewed, but limited toleration with explicit legal approval in the near future. At the time of the interviews the legal repercussions for GPs who continued to refuse to refer for abortions were not clear.

The 2011 regulation and the 2014 proposal to legally tolerate conscientious objection, have spurred debate. Opponents of conscientious objection claim that referrals cannot carry the ethical gravity of the actual performance of abortions, and that a right to conscientious objection to referrals could jeopardize women’s right to abortion [5]. Furthermore, it is feared that the doctor’s act of objection could be experienced by the patient as a violation and as moral condemnation [6]. Proponents claim that there ought to be tolerance for a moral minority when deeply held convictions are at stake, that referrals may be morally significant contributions to abortion, and that conscientious objection can be carried out in a way that safeguards the patient’s right to abortion [7].

A 2012 survey of 531 Norwegian medical students found that only 10% supported the right to conscientious objection to referrals for abortion [8]. The proportion increased to 32% for students for whom religion was important. 4.9% of the students – all citing high importance of religion – would themselves object to referrals. In 2013, after much debate, The Norwegian Medical Association came to support a limited right to conscientious objection, including objections to referrals, in matters "pertaining to life and death" [9].

To our knowledge, the present study is the first qualitative interview study of physician conscientious objectors. The study attempts to answer two main questions. First, when a GP refuses to refer for abortion, how does this actually take place? Second, how do the GPs justify their conscientious objection, and how do they square this practice with their professional duties towards patients? Through shedding light on this practice we also wanted to provide empirical data for subsequent normative analysis.

## Method

### Recruitment and characteristics of informants

Seven GPs were recruited for individual interviews. Recruitment was performed through requests to the Norwegian Christian Medical Association (NKLF), to which we believed that many objectors would belong. Two informants were recruited through the investigators’ own professional acquaintances. Ideally we would have wanted more informants, but repeated attempts did not yield more willing participants. However, it was decided not to include the GPs that had been most active and vocal in the public debate.

Of the seven informants four were women and three men. Their age ranged from 30 to 55, and they practiced in a variety of locations, ranging from cities to rural areas in western and central Norway. All informants were interviewed by the first author (EMKN) in their offices.

A question guide was used when conducting the interviews and modified after the initial interviews. In particular, informants were asked to describe in detail occasions on which they had refused to refer, their reasons for objecting and the perceived impact on physician-patient relationships. The interviews lasted about an hour, were taped and subsequently transcribed. Five interviews were conducted in the winter of 2012, whereas the remaining two took place in the first half of 2013.

### Content analysis

The analysis was performed by EMKN and MM independently, and in general followed the method of ‘systematic text condensation’ , four steps of content analysis developed by Malterud [10]. The method represents a feasible process for managing intersubjectivity and reflexivity in content analyses, while maintaining a responsible level of methodological rigour. We added a fifth step to this process, and analysed the empirical material using the following steps:

(1) From chaos to themes. The transcribed interviews were read to form an overall impression.

(2) From themes to codes. Units of meaning were identified and coded according to topic.

(3) From code to meaning. Each coded group was condensed and summarized in artificial quotations. At this stage several topics emerged; when the two independent analyses were later harmonized, these topics were condensed into five main topics and several sub-topics.

(4) From condensation to descriptions and concepts. The artificial quotations provided the basis for the development of the analytic text from which the final text of the article stems. Quotes from the interviews were translated from Norwegian to English during the writing of this article.

(5) From descriptions to interpretations. We continued our work until we felt we had reached an acceptable level of data saturation. We then compared quotations looking for both similarities that strengthened our impressions, and differences that weakened them [11].

### Research ethics

Informants were informed in writing and orally, and gave written consent. The project and the handling of recordings and transcriptions was approved by Personvernombudet (The Data protection office). According to the Norwegian system the study did not need institutional review board approval.

### Investigators’ preconceptions

As conscientious objection is a highly normatively charged issue, we believe that scientific investigators in this field do right in declaring their normative preconceptions. EMKN had no settled view on the morality of conscientious objection at the outset or throughout the study. HS supports GPs’ limited moral right to conscientious objection, with a pragmatic view on how these questions should be solved, so as not to interfere with the patients’ rights and the doctor-patient relationship. MM has authored academic papers on normative aspects of conscientious objection [12,13], supporting a limited moral right to conscientious objection that includes a GP’s objection to referrals for abortion. He has defended this view in the Norwegian public debate [7,14].

## Results

Five main topics emerged and will be presented in some detail: 1) carrying out conscientious objection in practice, 2) justification for conscientious objection, 3) challenges when relating to colleagues, 4) ambivalence and consistency, 5) effects on the doctor-patient relationship.

### How conscientious objection is carried out in practice

Among the respondents two main approaches emerged. The first group of GPs attempted to prevent all consultations with women seeking abortion from taking place. This they did by having their secretaries schedule all such consultations for one of the GP’s colleagues. The colleagues in question had explicitly agreed to this general arrangement.

For this first group of GPs the issue of referral for abortion nevertheless sometimes came up in the consultation, mainly because not all patients disclose their agenda to the secretaries. In such cases the GPs then immediately conveyed that they would not refer for abortion. The GPs expressed this in different ways, but were united in placing the emphasis on themselves, rather than on the (morality of) the act of abortion or factors pertaining to the patient. The GPs told the patient that they were unable to comply with the patient’s request for a referral. These GPs would then help patients set up an appointment with a colleague.

In this first group, only one GP had openly declared his conscientious objection in the local community. Subsequently this doctor had not had any consultations where a referral for abortion was requested. An additional two doctors had gained acceptance for their practices by the municipality at the time of appointment.

The second group of GPs wanted to have the consultations with the women requesting referral, and the doctors did nothing to prevent these consultations taking place. These GPs performed the physical examination, history taking, and gave information, but ultimately would not provide the referral itself. One doctor would immediately inform the patient that she would not provide the referral, whereas the others in this group did not convey their refusal before the end of the consultation. The doctors in this group expressed their refusal in ways similar to the first group. The patients were then informed about how to obtain a referral; typically the GP would arrange for contact with a colleague, who would then refer the patient.

Two of the informants in this second group worked in areas where the local gynecological department did not require a referral for abortion, but could accept patients directly. One of these GPs nevertheless made a point of stating to patients that she could not provide a referral. The other GP only disclosed her objection in the case that the patient requested referral documents. This GP maintained that her objection would be considerably more practically difficult in other parts of the country where a written referral is required.

Several of the informants pointed to another possible solution: a written statement that the patient was pregnant, as an alternative to a referral letter. The patient could then bring this statement to the gynecological department. These informants saw this alternative as morally preferable to writing a referral – a practical compromise that would satisfy both the patient’s rights and needs, and the GP’s own need for not contributing in the abortion process in a morally problematic way. However, none of the informants used this method more than sporadically.

Common to all informants was the emphasis on not unduly obstructing the fulfilment of the patient’s legal right to abortion. The informants also claimed that the requests for referrals for abortion were uncommon occurrences in their practices. With one exception the doctors had not informed their constituency about their objections, and neither did they see any need for this.

### Justification for conscientious objection, and associated emotions

#### Not contributing to taking a life

The informants maintained that unborn human life has moral value. Some explicitly stated that the embryo is valuable from conception. One informant’s expression was representative: ‘Human life is something very special, and we humans are not granted the option of taking a life’. All informants rooted this view at least partly in their Christian faith. Some also invoked the ethics of the profession: ‘I want to contribute to improving people’s lives, and to helping, soothing and comforting. Then it becomes self-contradictory to take lives’. Referral for abortion was portrayed as active participation in the process that leads to abortion. The informants emphasized the need to take responsibility for their own actions and contributions. Some informants had been opposed to abortion from their youth, whereas others had gradually changed their view of abortion towards a principled opposition. Similarly, some had refused to refer for abortion all through their careers as GPs, whereas for others the felt need to object had emerged gradually.

#### Unable to refer

Several described an inability to refer for abortions. If referrals had been demanded of them, these informants stated, they would not have been able to carry on as GPs. Some pointed to the importance of colleagues who could handle this task for them, and stated that they could not have been a GP in a rural setting without such colleagues. Two stated that they had referred on a few occasions, and that this lead to bad conscience and feelings of guilt. One said of this: ‘It felt like contributing to murder, in addition to breaking my own principles’. Two informants stated that they had previously lacked courage to refuse, but then had found that they could not handle referrals. One informant’s statement shows how the objection typically is tied to a Christian faith: ‘I am into spiritual counselling, I pray a little for people. I often place the hand that I write with on those I pray for. And then at one time it became clear that – I cannot sign this death sentence with the same hand that I use to bless’.

#### Being true to oneself

Informants maintained that having the opportunity to refuse referrals for abortion allowed them to be themselves. This was of great importance for most of our respondents. As one GP put it:

[The patients] meet actual persons during consultations. Being a doctor has a lot to do with the meeting between two or more people, and many come to you because *you* are the doctor […] personality is part of the package when you see the GP. And I also believe they want someone who talks back to them. I think it’s wonderful to be able to be allowed to be a whole person, to be allowed to be myself with my opinions.

Several also pointed out that patients appreciate that the GP is present with their own views and personality, and that patients profit from the GP giving his earnest view, sometimes also providing a little resistance. Several informants pressed that their refusals were not about communicating a stand against abortion, but were for the sake of protecting themselves and their own integrity. One GP stated:

With what I do, I do not have any missionary work in mind, I only have the thought of being able to survive as a doctor and a whole human being. My conscience is not a dress that I can put on or take off whenever I want to. My conscience must be there all the time, as a ballast, for me to remain a whole human being.

#### Uncertainty and respect for the choices of colleagues

Several stated that they were less than entirely comfortable with their chosen practice of refusals. Typically it was not seen as unproblematic, neither practically nor morally. A typical statement was, ‘There are many ways to do this, and I respect colleagues who choose differently’. Some also pressed that they were open to re-examining their current practice of refusals.

#### Inability to be neutral

Among the informants who avoided consultations with women seeking abortion, some stated that they would have liked to discuss the patient’s choice and options with them; however, the informants feared their opposition to abortion would preclude a stance of neutrality necessary for counselling. These informants thought that explaining their objections at the outset of a consultation and subsequently ending the consultation was necessary to avoid the impression of a moral condemnation of the patient.

The informants who performed the consultations with the women requesting abortion also thought it problematic to actively influence the patient’s choice. Rather, they described their role as aiding the patient in the decision-making through discussion and providing information. One informant said that he sometimes explained about fetal development, but would treat the topic sensitively, in order not to influence unduly. In addition, these informants thought that their potential influence on the patient’s decision was rather limited.

Some of the informants were careful to express respect towards the patient’s choice in the interviews. One said, ‘Even though I have a clear opinion about what I think is best, I feel humility towards the woman’s difficult choice. It is complex and in a way impossible’. Having to make such a choice was perceived to be very difficult.

### Relations to colleagues

The informants found their relations with their local gynecological departments to be unproblematic. As for their GP colleagues, the informants all had experienced understanding and respect for their views of abortion and their refusal practice. Even though some colleagues had spoken critically of conscientious objection in the media, our informants had not experienced negative reactions from colleagues they cooperated with daily. Two of the informants stated that their conscientious objection had been an entry point to good conversations with their colleagues about ethical dilemmas pertaining to abortion. The informants had all found colleagues who were willing to take over the consultations with the patients requesting abortion.

### Consistency

The informants were asked whether they saw their refusal practice as morally consistent. Informants saw challenges to their consistency along three axes: the reason for abortion, other issues regarding early human life, and degrees of cooperation in referral.

#### The reason for abortion

Most, but not all the informants agreed that the reasons underlying the abortion request mattered for their refusal to refer. These informants would have referred for abortion in cases of rape or incest, or when the mother’s life was in danger, and sometimes in other cases as well. One stated, ‘I referred to abortion for a woman who had two handicapped children from before. Her situation was so overwhelming, and to me it outweighed my own conscience’. Another stated, ‘I referred a young woman with an incredibly difficult social situation. This gave me feelings of guilt and was a violation of my principles, but I thought that referring this woman was something I had to do’. Some also stated that even though they would not want to partake in abortion themselves, they were in favour of the current abortion law which allows abortion on demand up to pregnancy week 12.

Only one informant stated that the reason for the abortion request would almost not influence his willingness to refer. He stated, ‘pregnancy due to rape would give me greater anguish than regular pregnancies, but I do not think I would have been able to refer. I think that life is sacred and inviolable, I have to take the consequences of this, and I am able to do that in 99 per cent of cases’.

#### Other issues regarding early human life

Several of the informants emphasized that abortion belongs in a spectrum of moral issues regarding early human life. Some thought that full consistency would require conscientious objection on other issues as well, such as prescribing contraceptives that may have post-fertilization effects, and referrals for in vitro fertilization in which spare embryos are created. However, most informants did not refuse in these cases. Their inconsistency was, however, perceived to be a necessary compromise. One GP stated:

I am not entirely principled or consistent in my actions. I prescribe contraceptives, and most of them can lead to abortions, even birth-control pills and hormonal IUDs. I have found a compromise I can live with. If you are to be truly principled, it is in reality incompatible with being a GP. (…) If I were to object to IUDs, I had to object to the pill and other contraceptives as well, and that would be a bit too much. I have used the pill myself, and I think it would be contradictory [to oppose it]. If I object to contraceptives, then the women would have to return for an abortion a few weeks later.

One informant would refuse to refer for assisted reproduction for same-sex couples. But this was a decision he would be willing to reconsider, stating that ‘that argument does not run so deep with me’. Other informants did not think that their practice involved ethical inconsistency.

#### Degrees of cooperation in referral

Several informants reflected on the moral value of their chosen way of objecting to referrals. Did the actions they took to ensure that the patient’s right to treatment was fulfilled (e.g., ensuring that the patients were seen by colleagues) implicate the informants in morally culpable cooperation? Informants had no definitive answer to this. A typical statement was,

Then there is the question of where the line goes for what one ought or ought not to do. With the two or three I have had I have said that they can go to the gynecological ward without a referral letter. I could have refrained from saying that. (…) It is really an artificial line – I have put the line at not signing my name.

### Burdens to patients and challenges to the physician-patient relationship

#### Refusal to refer as a potential rejection of the patient

A few GPs thought that their refusals could be experienced by the patient as a kind of rejection, causing disappointed patients, and perhaps damaging the doctor-patient relationship. One GP stated:

It is a problem, it does something to the relation with the patient – that I do not provide care fully, I feel that (…) I fail them a little bit in not taking part in the entire course – this is something that is harmful for the physician-patient relationship.

The informants assessed that physician-patient relations in the majority of cases had not been negatively influenced by the GP’s refusal. The informants all thought that it was unproblematic for them to relate to the patients concerned in future consultations. A few stated that they treated some of these patients for post-abortion grief in the aftermath of the abortion.

Some informants also stated that it is of the nature of the GP’s job to sometimes decline the patients’ requests. For instance, requests for certain diagnostic procedures, referrals, or sick leave certificates must sometimes be turned down. One GP stated, ‘As a GP … people come to us with many wishes we cannot fulfil, asking for sick leaves (…) and it is [often] completely out of the question. That is also a rejection.’

Furthermore, a few informants also stated that they had experienced understanding and acceptance for their attitude towards abortion referrals from the patients concerned. One stated, ‘I think people here have great respect for the fact that people have opinions. That is my explanation for why I have not encountered greater resistance’.

#### An established physician-patient relationship as a safeguard against the experience of rejection

Several informants pressed the point that a well-established and long-running physician-patient relationship would make the GP’s refusal to refer easier for both doctor and patient. One GP stated:

Our advantage [as GPs] is that we know patients for years. We have built a relationship in advance, which does not break just because of something that is not quite perfect. Refusals will be harder with a patient you do not know.

Some GPs said they had discussed just about everything with their patients, so a refusal to refer would not really mean a rejection. One GP stated that she was sure that a patient she had known for years saw her refusal as ‘just a parenthesis’ in their relationship.

#### Burdens to patients

Apart from the potential experience of rejection, mentioned by some informants, the informants did not think that their refusals led to burdens of significance for patients. One emphasized that although the patients do not receive the referral they want from their GP, they do receive proper health care as defined by the law, through being referred from another GP or being able to contact the gynecological ward directly. One informant stated, ‘I do not think my practice here is any more blameworthy than that I am not up-to-date on all medical fields at all times – which I cannot be as a doctor’. Speaking of a particular patient, the informant said: ‘If one views it as objectively as possible then I do not think she has suffered any burdens – the only potential burden must be to hear that others have different views on the principled aspects of her choice’.

## Discussion

### Consistency, ambivalence, and absolutism

According to a 1999 study on moral attitudes, Norwegians tend to separate between the questions "what is morally right and wrong", and "what should be allowed in a legal sense" [2]. E.g., in 2004, more than 50% of Norwegian women who considered themselves conservative on religious questions, supported the right to abortion on demand [15]. The 1999 study further showed that Norwegian doctors are more permissive toward abortion (which is legal) than the general population, but less permissive regarding euthanasia (which is prohibited). This could indicate that Norwegian doctors subscribe to a separation of law and morality to an even greater extent than the general population.

In a study among health professionals working in prenatal screening, Farsides et al. introduced the labels ‘facilitators’ , ‘tolerators’ , and ‘absolutists’ [16]. Whereas facilitators separate their own moral views from their professional duties, and tolerators accept the perceived wrongdoing of another, absolutism ‘entails having a moral belief about something which is fixed and non-negotiable’.

One would perhaps expect conscientious objectors to be absolutists in this respect, but the label does not fit our informants entirely. Most displayed ambivalence towards situations in which an abortion was thought to be especially well justified, and some did in fact refer for abortion in such situations. In addition, many informants thought that their practice was not fully consistent when other issues pertaining to early human life were considered, and that their practices amounted to a degree of cooperation that was a necessary compromise.

Thus, even though the category of the absolutist is helpful for theoretical delineation of different attitudes, the typical absolutist – at least among our informants – is not *absolutely* absolutist, but rather marked by a striking ambivalence and a willingness to make certain compromises (e.g., prescribing contraceptives that have post-fertilization effects). A certain pragmatism seems to prevail, even among most doctors not willing to make referrals to abortion.

### Diversity of refusal practices, lack of legal regulation, and christian denominations

The study uncovered a diversity of practical arrangements among conscientious objectors. Informants differed as to whether they attempted to avoid the consultations with the patients seeking abortion, as to at what stage in the consultation they communicated their objection, and as to how they ensured the patient’s subsequent access to abortion. They also differed as to whether they had informed officials in the municipality in which they practiced, and whether they had informed the patient population they served – something only one of the informants had done.

We identify two factors that we think can contribute to an explanation of this observed diversity: the relative lack of a developed legal regulation of conscientious objection; and the lack of guidance from Christian denominations. First, the relative ‘legal vacuum’ (until 2011) gave the GPs an incentive to conduct their refusals ‘silently’. If these practices were to surface and become a public concern, this could potentially lead to legal repercussions and explicit societal disapproval. The legal vacuum meant that no guidelines for how referrals ought to be conducted were available. The silent refusals also meant that conscientious objectors were unlikely to know many like-minded colleagues. In sum, each physician was left to figure out for themselves *whether* and *in which situations* to object, and *how to go about* objecting.

Second, Protestant and other non-Catholic churches, to which five of the seven informants belonged, typically do not issue detailed guidance in bioethical questions. For instance, the Church of Norway (the former state church) has no settled view on how physicians should deal with abortion. If no guidance was forthcoming from legal rules, then, neither was there any from official church bodies. Again, conscientious objectors were left to shape their practices on their own.

However, the two remaining informants were Roman Catholics. For one of these, but not for the other, the Catholic Church’s official stance on cooperation in abortion was important. This informant stated that obligations towards the church’s teaching were crucial in guiding and shaping the line of action chosen.

As a consequence of refusal practices surfacing and becoming a public concern, public attention has led to debate on how refusals ought to be carried out in practice. If official regulations are issued, these may prescribe the practical arrangement for refusal practices that the public and the authorities deem most appropriate.

### Well-established physician-patient relationships and the acceptability of refusals

In the normative literature on conscientious objection it is sometimes stated that conscientious objection is only – or at least more – morally acceptable if it takes place before a physician-patient relationship has been fully established [17]. The main reason is that the physician in such cases has not yet promised the provision of health care services.

However, a novel argument to the opposite effect may be constructed from the present findings. Many of the informants had the impression that a long-standing physician-patient relationship was relatively impervious to deleterious effects of disagreements and moral differences. In a well-established relationship the physician and the patient know each other well and are, seemingly, likely to be more tolerant of each others’ idiosyncrasies. Patient-doctor relationships are generally more trustful when they have lasted for a few years [18]. The Norwegian ‘regular GP scheme’ facilitates the establishment of long-running physician-patient relationships.

By and large the informants did not think that their practice involved significant burdens to patients. However, the objecting GPs’ own reports of course do not settle this issue (see Limitations below). Many patients seeking abortion are young women who may not have formed long-running relationships with their GPs. They may feel more abandoned by the GPs’ refusals than the GPs themselves claim in our interviews.

In the GP’s refusal may lie the potential for negative patient experiences of rejection and moral condemnation. In the Norwegian debate, this potential for negative experiences has figured prominently as a weighty argument against conscientious objection. This is in contrast with the international academic debate, in which this argument typically is not mentioned or lent much weight [17,19,20]. We believe this observation can be partly explained by objections in Norway often occurring ‘silently’. The practice is not transparent, and so when patients confront a GP who refuses to refer, they may be wholly unprepared. Another factor that can help explain the significance given to the potential for rejection experiences is that abortion is considered to be a settled issue by most (i.e., a right to abortion on demand is accepted by a large majority); patients may thus be unprepared for encountering a moral dissident at the doctor’s office.

### Performing versus referring, and varieties of referrals

Granted that refusals to perform abortion are ethically justified, how strong is the corresponding justification for refusals to refer? Our informants do not address this question directly, but are unanimous in perceiving referrals as ethically highly problematic active participation in the abortion process. Still, the informants saw it as a duty not to obstruct the continuity of care. They had all arranged for colleagues to take over their consultations.

Our findings include nuances about referrals that the normative literature often does not take into account. Typically, the literature discusses the moral complicity involved in referring to an abortion clinic (‘vertical referral’), and in referring to a colleague who then refers for abortion (‘horizontal referral’). In addition, Chervenak and McCullough discuss ‘indirect referrals’ , thereby designating the act of merely ‘providing patients with referral information’ [21]. Authors have differing views on the moral acceptability of the complicity involved in various kinds of referrals [17,21,22].

However, our findings indicate that horizontal referral can be carried out in different ways. For instance, horizontal referral can be both the act of personally ensuring that the patient is offered a consultation with a colleague who then refers; and developing systems that will ensure that patients go directly to a colleague, thus bypassing the conscientious objector entirely. In addition, a written confirmation that the patient is pregnant was seen by some as a (morally preferable and distinct) alternative to a direct referral. It becomes a question for normative analysis whether different varieties of horizontal referral and the actions involved therein constitute different degrees of moral complicity, and whether any differences are of moral import.

### Different refusal practices may make a moral difference

A normative analysis of the diverse refusal practices uncovered in this study may find that the practices are not necessarily morally consistent. This is a discussion we will not enter into here, other than pointing out some possible questions for subsequent normative analysis. For instance, Wicclair argues that the moral justification for conscientious objection is strengthened to the extent that the doctor seeks to minimize burdens to patients [17]. Which practical arrangement will burden patients the least – the advance disclosure to the patient population, the secretary’s direct arrangement of a consultation with a colleague, the GP’s disclosure of their refusal at the outset of the consultation, or the disclosure at the end of the consultation? And are the differences of moral significance?

One upshot is that there may be room for professional guidelines for how conscientious refusals should take place in practice. That is, if society deems that one way of conducting the refusal is more acceptable than others, and that this way is in fact acceptable, then a practical guideline for conscientious objectors could shape the way refusals take place, ultimately minimizing burdens to patients. For instance, a guideline could demand advance notification of the GP’s objections for the patient population.

### The study’s weaknesses

The study includes fewer informants than would be ideal. However, the study has merit in that each participant provided rich descriptions, and in shedding light on an under-researched topic. All informants were Christians; however, it is generally believed that most if not all Norwegian GPs who object to referrals for abortion are in fact Christians. The interviews gave the informants the opportunity to present their practices in a positive light. The informants can have had an incentive for a positive self-representation, especially considering the public controversy surrounding conscientious objection. When interpreting the informants’ reports it is vitally important to keep in mind that these reports, however illuminating, do not give the full picture of the practice of conscientious objection. Crucially, the patients’ own views are missing here. How do patients seeking referral for abortion experience their GP’s conscientious refusal? Very little is known about this, and there may be methodological obstacles to the empirical study of these experiences. For the assessment of patients’ experiences we currently only have indirect sources such as media stories and complaints to the health authorities that have been made public.

## Conclusions

The study uncovered a diversity of practical arrangements for refusals to refer for abortion. A striking find was the physicians’ ambivalence and what they themselves perceived as ethical inconsistencies. The protection of moral integrity was perceived as vital; at the same time, informants wanted to ensure that the patient’s interests and the physician-patient relationship were not harmed. Conscientious objection to referral yet – through arranging for a consultation with a colleague – assuming a certain responsibility for the patient’s access to abortion services, was seen as a practically feasible and morally acceptable compromise. In short, conscientious refusals were pragmatic solutions to a moral dilemma.

## Competing interests

MM has defended the GP’s limited right to conscientious objection in public debates.

## Authors’ contributions

MM and EMKN designed the study. EMKN conducted and transcribed the interviews. EMKN and MM analysed the interviews separately, then drafted the article together. HS gave methodological advice and contributed to analysis, background information, discussion and article revision. All authors approved the final manuscript.

## Pre-publication history

The pre-publication history for this paper can be accessed here:

http://www.biomedcentral.com/1472-6939/15/15/prepub
